# Patterns and implications of spatial covariation in herbivore functions on resilience of coral reefs

**DOI:** 10.1038/s41598-024-83672-1

**Published:** 2025-01-07

**Authors:** Dana T. Cook, Sally J. Holbrook, Russell J. Schmitt

**Affiliations:** 1 Department of Ecology, Evolution and Marine Biology, University of California Santa Barbara, Santa Barbara, CA 93106 USA; 2 Coastal Research Center, Marine Science Institute, University of California Santa Barbara, Santa Barbara, CA 93106 USA; 3 Present Address: One People One Reef, Santa Cruz, CA 95073 USA

**Keywords:** Herbivory, Reversal of state shifts, Grazing, Browsing, Community recovery, Vulnerability and reversibility, Community ecology, Community ecology

## Abstract

Persistent shifts to undesired ecological states, such as shifts from coral to macroalgae, are becoming more common. This highlights the need to understand processes that can help restore affected ecosystems. Herbivory on coral reefs is widely recognized as a key interaction that can keep macroalgae from outcompeting coral. Most attention has been on the role ‘grazing’ herbivores play in preventing the establishment of macroalgae, while less research has focused on the role of ‘browsers’ in extirpating macroalgae. Here we explored patterns, environmental correlates and state shift consequences of spatial co-variation in grazing and browsing functions of herbivorous fishes. Grazing and browsing rates were not highly correlated across 20 lagoon sites in Moorea, French Polynesia, but did cluster into 3 (of 4) combinations of high and low consumption rates (no site had low grazing but high browsing). Consumption rates were not correlated with grazer or browser fish biomass, but both were predicted by specific environmental variables. Experiments revealed that reversibility of a macroalgal state shift was strongly related to spatial variation in browsing intensity. Our findings provide insights and simple diagnostic tools regarding heterogeneity in top-down forcing that influences the vulnerability to and reversibility of shifts to macroalgae on coral reefs.

## Introduction

A major focus in ecology is to understand the complex dynamics of ecosystems that simultaneously are being buffeted by rapidly changing disturbance regimes and by more slowly changing environmental drivers. Of considerable interest are abrupt transitions between qualitatively different ecosystem states^1,2^, which can be challenging to anticipate and problematic to reverse^3,4^. The consequences to society of such shifts can be profound if an alternative community state provides either fewer or qualitatively different ecosystem services^3,5^. This adds urgency to the need to better understand the processes that influence transitions between alternative, self-reinforcing states (regime shifts). In general, shifts in state can occur when the magnitude of a perturbation overwhelms the internal resilience of a system, which itself can be eroded when key biological processes are altered by human activities or natural events. For example, climate change can both reduce the interval between fire disturbances and alter the demographic rates of plants, which can result in a regime shift in some forest ecosystems^6^. Resolving links between state shifts, biological processes, and ecosystem resilience can inform management strategies intended to maintain desired ecosystem states during this era of unprecedented human impact on the environment^7^.

Coral reefs are being degraded worldwide by local and global stressors and acute disturbances^8–10^. Declines in coral cover caused by storms, predator or disease outbreaks, or bleaching frequently have been accompanied by concomitant increases in macroalgae (coral-algae ‘phase shifts’), as has been reported in the Caribbean^11^, the central^12^ and western Pacific^13^, and the Indian Ocean^14^. Macroalgae generally are superior competitors to coral and can preclude a return to a coral-dominated state even if sufficient coral propagules are present^8,15^. Top-down control of macroalgae by herbivores is widely recognized as a crucial biological process that can influence shifts between coral and macroalgae^4,8,13,15–20^. In this context, despite a broad range of feeding strategies, mobile herbivorous fishes on coral reefs have two general functions^13,20–25^, which we operationally term ‘grazing’ and ‘browsing’ (e.g.,^25^). Following a coral mortality event, grazing herbivores that consume endolithic and filamentous algae *prevent* the establishment of macroalgae by consuming their early developmental stages on the disturbed substrate, thereby maintaining the reef surface in a condition (cropped turf) that can be colonized by coral^13,15,20,25–29^. However, if macroalgae do become established on the reef, they subsequently can be removed by species of browsing herbivores that consume mature macroalgae^13,20,25,28,30^. Thus, browsers, but not grazers, play a critical role in *reversing* coral-to-macroalgae state shifts and enhancing the potential for reestablishment of coral^31^.

It has been posited that having robust populations of both grazing and browsing species contributes to coral reef resilience^13^, a notion that modeling of these systems has supported^32^. Evidence suggests that grazers typically have higher species richness and biomass than browsers on tropical reefs, both at local reef tract^15,25,33^ and larger scales^13,30,34–37^. This pattern can be exacerbated on reefs where local-scale fisheries heavily target browsing species^32,36,38^, potentially lessening the chance of a state shift reversal from macroalgae back to coral. For example, in the local-scale lagoon fishery in Moorea, French Polynesia, browsing unicornfishes (*Naso* spp.) are highly prized^39^ and targeted^36^. Models indicate that selective fishing of these browsers can push the system close to a tipping point from coral- to macroalgal-dominance^32^. Further, persistent shifts to macroalgae at some locations on the Great Barrier Reef have been correlated with low abundances of browsing fishes^13^.

Knowledge of the spatial patterns of covariation in herbivore grazing and browsing functions might provide insight into both the vulnerability of a reef to a coral-to-macroalgae state shift as well as the potential for such a shift to be reversed. There has been an emphasis in prior research and management approaches on the maintenance of coral dominance to prevent a shift to macroalgae, and less on the reversibility of such a shift to re-establish coral dominance^28,31^. Here we address that information deficit by exploring patterns and environmental correlates of spatial covariation in grazing and browsing functions of herbivorous fishes, and relating those patterns to both the vulnerability to and potential reversibility of a coral-macroalgae phase shift. The lagoons of Moorea, French Polynesia, provide an ideal model system to explore spatial variation in herbivory functions and the potential consequences of that spatial pattern to coral resilience at the local patch reef scale. Experimental studies conducted in Moorea suggest coral and macroalgae can be alternative basins of attraction (i.e., represent a regime shift,^4,15^), and accompanying time series data and other process studies show that herbivory has been essential to coral recovery following large mortality events^17,18,40^. Here we quantified a range of biological and physical characteristics (rates of grazing and browsing, biomass and taxonomic composition of the herbivorous fish community, benthic community composition, productivity of algal turf and of macroalgae, nutrient enrichment and proximity to the reef crest and deep-water channels) at 20 sites. These data were used to explore spatial patterns in herbivory within the lagoons. Further, ‘hot spots’ and ‘cold spots’ that had either high or low levels of herbivory respectively were identified and the environmental attributes of each type of site were characterized. In the coral reef literature, there is a paucity of experimental attempts to quantify the potential for reversibility of the macroalgae state (but see^28,31^). We explored this using a field experiment conducted along a natural gradient in browsing intensity to evaluate whether reversal potential was related to variation in browsing intensity. Our findings have considerable relevance to the development of spatially-explicit management actions to enhance resilience of coral reefs.

## Results

### Patterns of spatial variation in rates of grazing and browsing

**Fig. 1:**
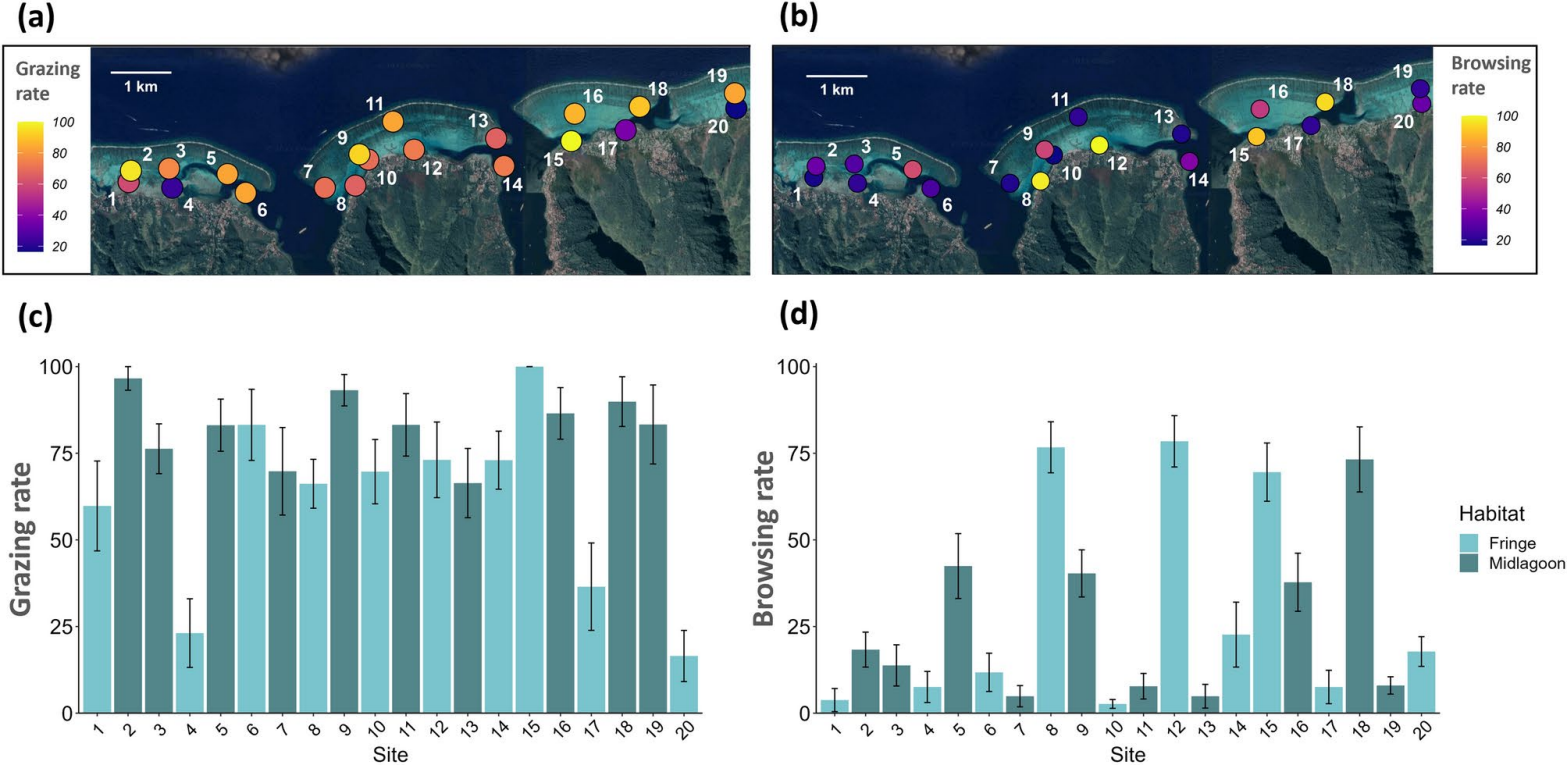
Maps showing percent consumption (mean ± SE) of (**a**) turf assays after 3 h (N = 10 replicates per site) (i.e., grazing rate) and (**b**) macroalgae assays after 24 h (N = 15 replicates per site) (i.e., browsing rate) across the twenty sites. Yellow denotes high rates of herbivory and blue indicates low rates. Bar plots show the same rates of (**c**) grazing and (**d**) browsing at the twenty sites ordered from west to east. Bars are colored according to reef habitat.

**Fig. 2:**
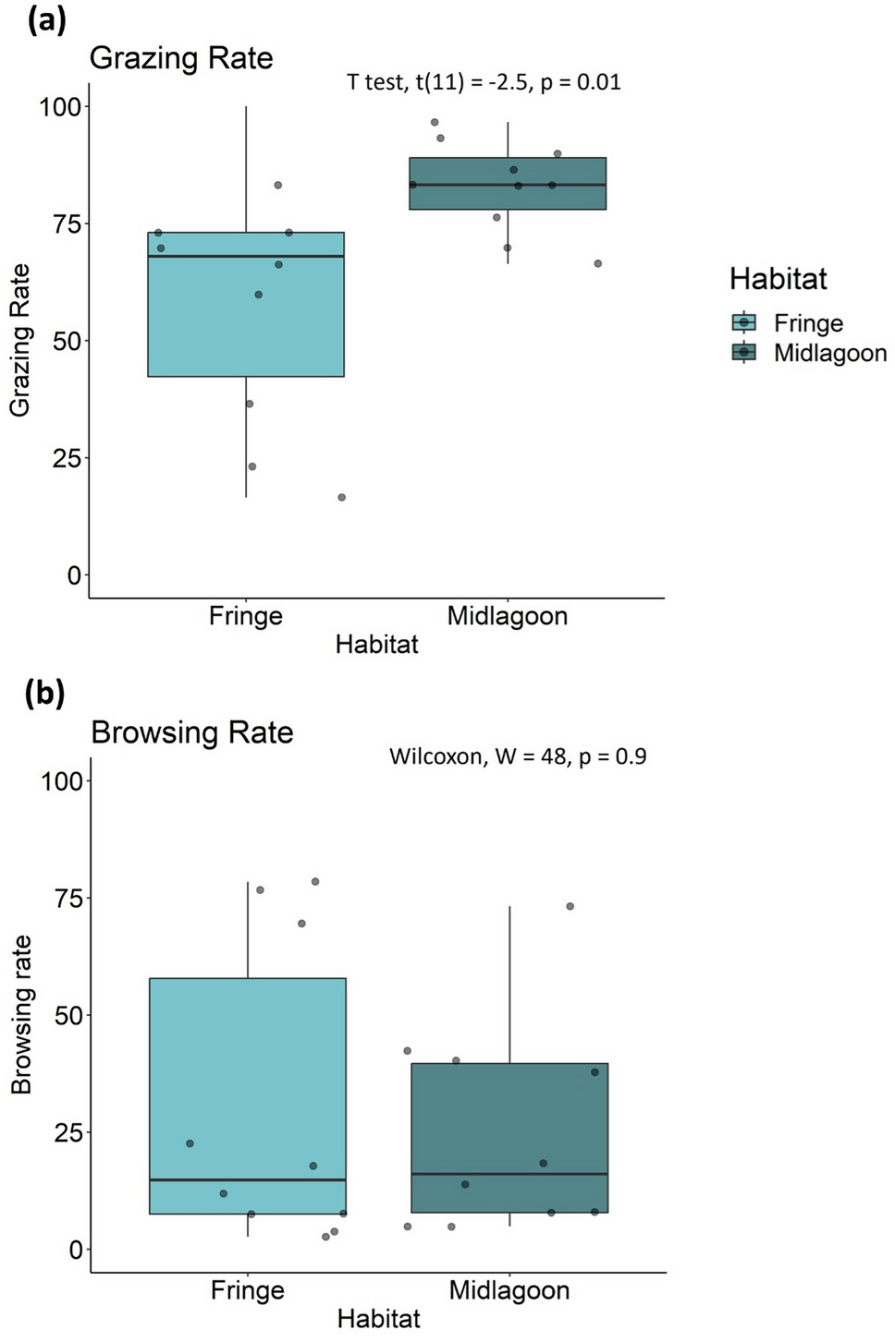
Boxplots showing rates of (**a**) grazing and (**b**) browsing at the twenty sites grouped by habitat (N = 10 sites per habitat). Black lines within boxes are medians in grazing or browsing. Lower and upper box boundaries are 25th and 75th percentiles, respectively. Grey circles indicate the average rate of grazing or browsing at a site.

Assays to estimate intensity of grazing on algal turf and of browsing on macroalgae were conducted at 20 study sites in the lagoon (SI Fig. 1). We found no evidence of spatial autocorrelation in grazing or browsing intensity across the 20 sites (grazing: Moran’s I = − 0.05, *p* = 0.5; browsing: Moran’s I = − 0.05, *p* = 0.5). Assays revealed high spatial variation in herbivory rates across the lagoon reef system (Fig. 1). Of the sites examined, many had moderate to high levels of grazing, and only a few had low levels of grazing activity (Fig. 1a,c). By contrast, many sites were characterized by low or moderate levels of browsing, with few having high browsing activity (Fig. 1b,d). We first explored patterns in grazing and browsing along two spatial gradients: (1) alongshore (west to east along the north shore), and (2) cross-shore between two major reef habitats – fringing reefs and mid-lagoon reefs. No alongshore trend was apparent in either grazing or browsing (Fig. 1c,d). However, grazing – but not browsing – differed between habitats; grazing was higher on mid-lagoon reefs than fringing reefs (grazing: *t*_(11)_ = 2.5, *p* < 0.05), browsing: Wilcoxon’s test W = 48, *p* = 0.9) (Fig. 2).

**Fig. 3:**
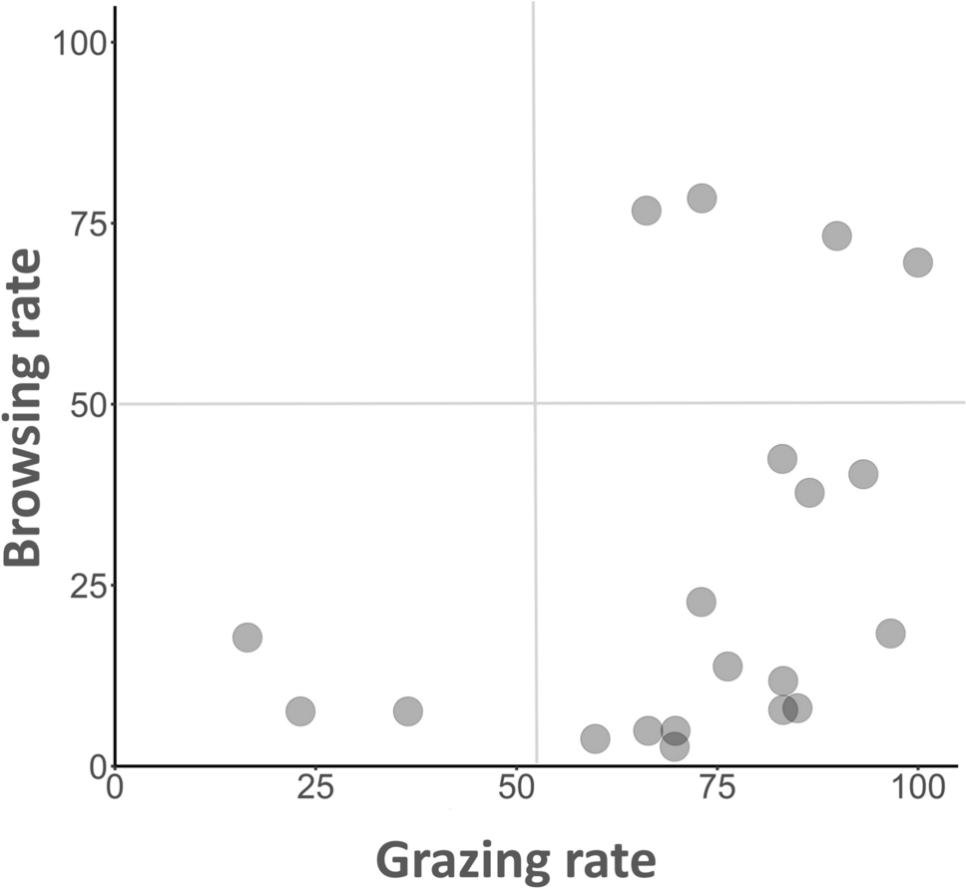
Spatial covariation between grazing and browsing rates across the twenty sites. Values are the site averages shown in Fig. 1. Grazing rate is mean percent consumption of turf assays after 3 h and browsing rate is mean percent consumption of macroalgae assays after 24 h.

Grazing and browsing rates were not spatially correlated with each other across the twenty sites (Pearson’s *r* = 0.36, *p* = 0.12). Despite this lack of concordance, a striking spatial pattern was apparent (Fig. 3). Sites clustered into three of the four possible herbivory regimes (i.e., the orthogonal combinations of high and low rates of grazing and browsing), which were: (1) low grazing-low browsing, (2) high grazing-low browsing, and (3) high grazing-high browsing (Fig. 3). None of the sites were characterized by low grazing but high levels of browsing (Fig. 3).

### Relationships between spatial variation in herbivory, herbivore biomass, and environmental attributes

**Fig. 4:**
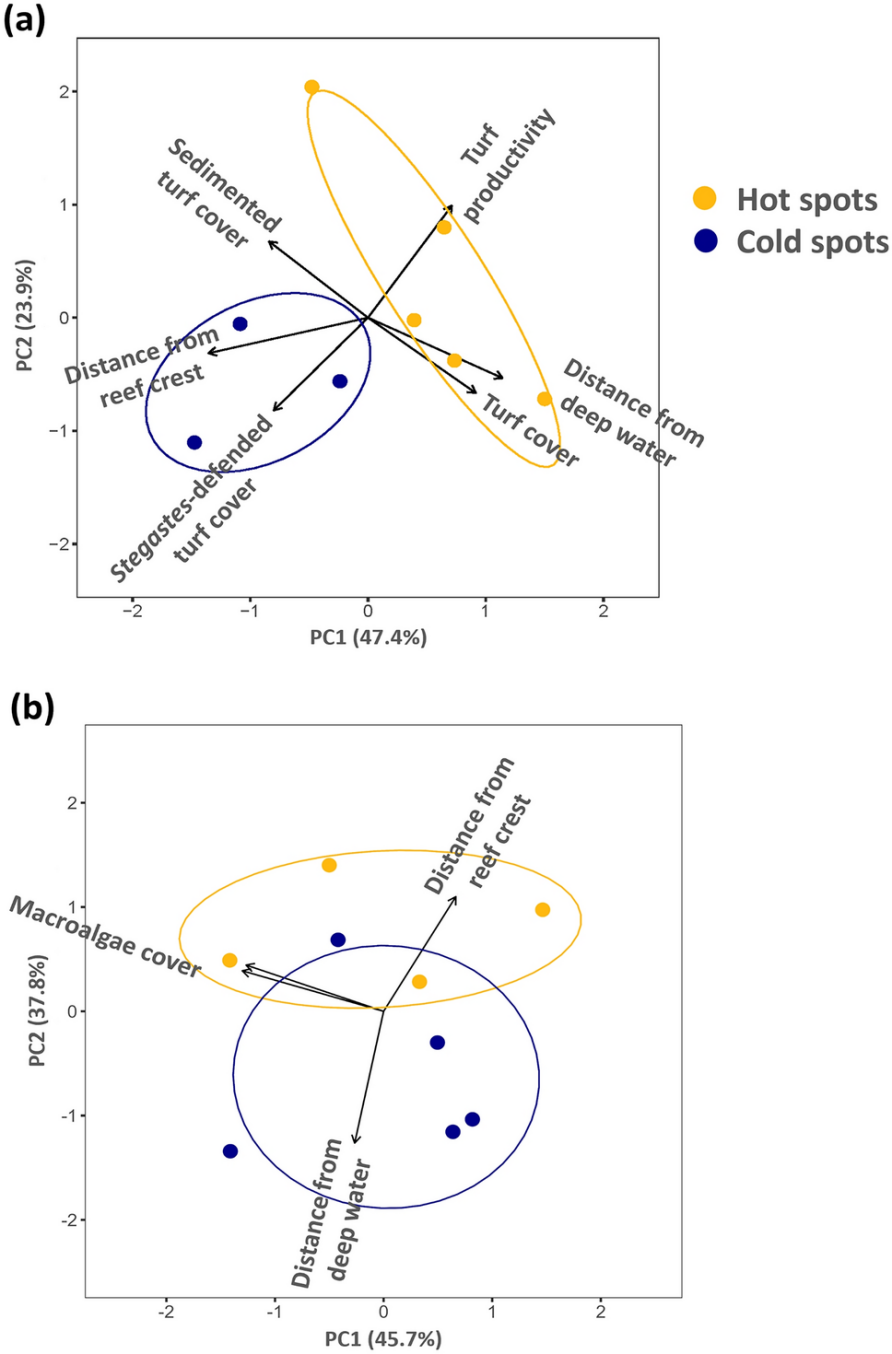
Principal component analysis showing the relationships between spatial variation in (**a**) grazing and (**b**) browsing with environmental attributes at hot and cold spots (i.e., sites with high or low herbivory rates). Yellow circles: hot spots in grazing (N = 5 hot spots) or browsing (N = 4 hot spots). Blue circles: cold spots in grazing (N = 3 cold spots) or browsing (N = 5 cold spots).

Principal component analyses (PCA) explored the degree to which environmental factors [cover of macroalgae and of different types of turf (cropped turf, sedimented turf, *Stegastes*-defended turf cover), productivity of algal turf and of macroalgae, nutrient enrichment, and geographic location (proximity to the reef crest and deep-water channels)] could explain observed spatial patterns in browsing and grazing, specifically, the ‘hot spots’ (high grazing or browsing activity) and ‘cold spots’ (low activity) in the lagoon. A subset of sites that reflected the highest (hot spots) and lowest (cold spots) levels in each of the herbivory processes was selected for analysis. We found that different sets of variables explained the variance for each aspect of herbivory. Grazing ‘hot’ and ‘cold’ spots (i.e., high and low rates respectively) separated in ordination space along both axes PC1 and PC2, with cumulative proportion of variance explained = 0.71 (Fig. 4a). Loading revealed that spatial variation in grazing was related to turf productivity, cover of the different categories of turf, and geographic location. Grazing hot spots were characterized by high productivity and cover of cropped turf as well as increased distance away from deep water drop-offs and closer proximity to the reef crest. Conversely, grazing cold spots were associated with turf containing high sediment loads, high cover of turf algae gardens defended by farmerfish (*Stegastes*), increased distance from the reef crest and closer proximity to deep water drop-offs.

By comparison with grazing, the PCA for browsing revealed that hot and cold spots separated almost entirely along PC2, which was associated with geographic location (Fig. 4b; cumulative proportion of variance explained for Axes 1 and 2 = 0.84). Loadings revealed that browsing hot spots tended to be further from the reef crest and closer to deep water drop-offs, whereas cold spots were generally the converse—at sites closer to the reef crest and farther from deep water.

Patterns of grazing were associated with differences in the composition of turf communities between the mid-lagoon and fringing reef habitats. Mid-lagoon sites, which consistently had high grazing rates, were characterized by high cover of cropped turf that contained little or no sediment (Wilcoxon’s test = 21, *p* = 0.03, SI Fig. 2a). Fringing reefs, which supported generally lower and spatially more variable levels of grazing, were characterized by higher cover of turf with high sediment loads (*t*_(3)_ = 2.7, *p* = 0.008, SI Fig. 2b) and turf gardens defended by the farmerfish *Stegastes* (*t*_(3)_ = 2.5, *p* = 0.02, SI Fig. 2c).

Visual surveys of the herbivorous fishes conducted by divers at the 20 study sites revealed a total of 19 species. As expected, browsing species were less speciose in the assemblage (4 of 19 species) and they comprised only about three percent of the total biomass and of the total abundance (SI Table 1). Browsers included species in 3 different genera (*Leptoscarus*, *Naso*, *Siganus*). Grazers were represented by 5 genera, with *Acanthurus* and *Scarus* predominant with 7 and 4 species respectively (SI Table 1).

We did not find the observed spatial variation in our measured rates of grazing or browsing was correlated with our visually-based estimates of fish herbivore biomass among the sites (SI Fig. 3). Neither the among-site variation in the measured rate of grazing (Pearson’s *r* = 0.21, *p* = 0.37, SI Fig. 3a) or browsing (Pearson’s *r* = − 0.24, *p* = 0.3, SI Fig. 3b) was related to the estimated local biomass of fish grazers or browsers, respectively.

### Predicting variation in herbivore biomass using environmental attributes

Despite the fact that herbivore biomass and herbivory rates were not strongly correlated, spatial variation in the biomass of each herbivore group did map onto the same major environmental variables relating to the spatial patterns in grazing and browsing as revealed by our PCA analyses (SI Table 2). An AIC-based model selection multiple linear regression indicated that variation in grazer biomass across the twenty sites was best predicted by turf productivity and, more weakly, cover of sedimented turf, which together explained 24% of the among-site variation in grazer biomass (*F*_(2, 7)_ = 3.96, *p* = 0.04). The best model for browsers revealed that variation in macroalgal productivity and distance from the reef crest were the best combination of predictors, which together explained 36% of variation in browser biomass (*F*_(2, 17)_ = 6.28, *p* = 0.009).

### Linking spatial variation in herbivory to the resilience of coral

**Fig. 5:**
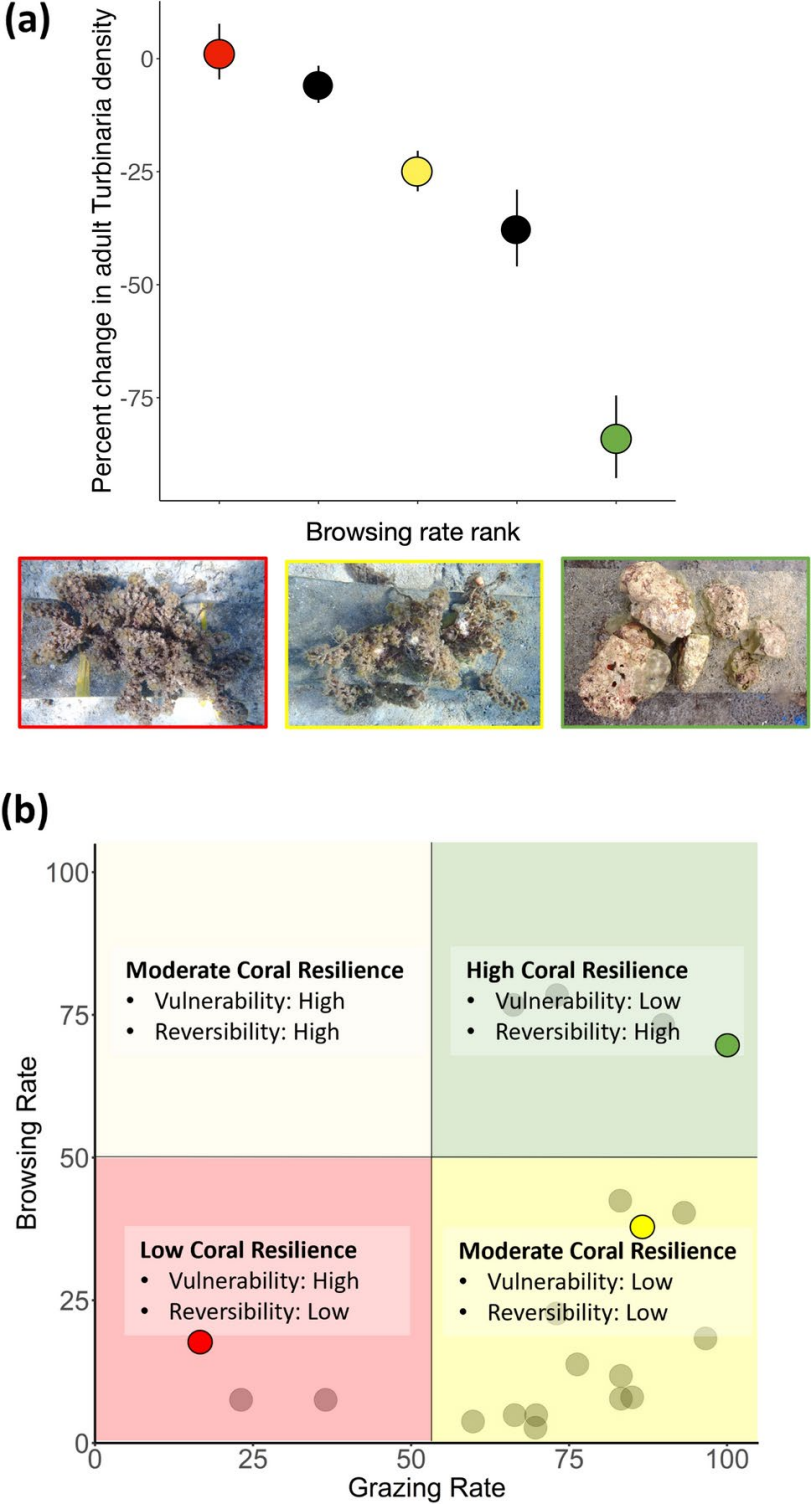
(**a**) Percent change (mean ± SE) in adult *Turbinaria* density within macroalgal communities (N = 6 plots per site) along a gradient in ambient browsing (based on short-term browsing assays; sites ordered from lowest to highest levels). Three of the sites were chosen from the original set of twenty sites. Their location on panel b is noted using the same color. (**b**) Conceptual framework linking variation in herbivory to vulnerability to and reversibility of coral-to-macroalgae state shifts. Vulnerability to shifting to macroalgae is tied to grazing level; sites with low grazing are highly vulnerable to shifting to macroalgae (tan and red quadrants) and those with high grazing will likely remain in a cropped turf state (green and yellow quadrants). Reversibility of a coral-to-macroalgae shift is tied to browsing level; if macroalgae establish, sites with low browsing are likely to remain in a macroalgae state (i.e., low reversibility –yellow and red quadrants) and sites with high browsing could return to the coral state (i.e., high reversibility – tan and green quadrants).

Because of a general lack of data that link browsing intensity and reversibility of state shifts to macroalgae, we explored this relationship using a field experiment. Macroalgae-dominated experimental units were exposed to herbivores along a natural gradient in browsing intensity (determined by herbivory assays) to evaluate whether reversal potential was related to variation in browsing. Spatial variation in reversibility was strongly related to browsing intensity; the capacity of browsing herbivores to remove late successional communities of macroalgae increased along a gradient in browsing (Fig. 5a). After 10 days, the average density of adult *Turbinaria* within experimental communities in low browsing areas either did not change or decreased by only 6% (Fig. 5a). This implies reefs in areas with low browsing capacity are vulnerable to remaining trapped in a macroalgae-dominated state (Fig. 5b yellow and red quadrants). By contrast, herbivores in the high browsing site removed 84 ± 9% (mean ± SE) of *Turbinaria* and in some plots completely removed macroalgal communities, suggesting high potential for reversibility if a shift were to occur (Fig. 5b tan and green quadrants). We found that reversibility significantly increased with closer proximity to deep water (Pearson’s *r* = 0.89, *p* = 0.044, SI Fig. 4), which paralleled the pattern observed for spatial variation in browsing intensity revealed by our short-term assays (Fig. 4b).

## Discussion

Grazers and browsers are critical for preventing and reversing state shifts on coral reefs, respectively, and there is concerning evidence that small-scale fisheries may disproportionately reduce browser biomass relative to grazers. Given the posited implications of such a disparity in functional impact of these two herbivore groups for coral-to-macroalgae state shifts^13,15,32^ it is crucial to better understand spatial patterns of grazing and browsing and how variation in these processes impacts the resilience of coral. Here, we investigated spatial variation in grazing and browsing, identified key environmental factors contributing to that variation, and assessed how covariation in these distinct herbivory processes may influence resilience of the coral state in a system where coral and macroalgae have been demonstrated experimentally to be alternative basins of attraction under certain environmental conditions^4,15^.

Our study revealed high spatial variation in herbivory rates across a ~ 10 km stretch of the lagoons of the north shore of Moorea (Fig. 1, SI Fig. 1) with different patterns emerging for grazing and browsing. Hot and cold spots in grazing or browsing could be as close in space as a few hundred meters (Fig. 1). Grazing hot spots were more common across lagoons than browsing hot spots, possibly reflecting the disparity in species richness and abundance of grazers and browsers in Moorea (SI Table 1). Although we did not detect alongshore or cross-shore gradients in browsing, we found grazing varied between fringing and mid-lagoon reef habitats that differ in distance from shore. Rates of grazing were consistently high on mid-lagoon reefs but variable on fringing reefs. This decline in grazing impact across a reef gradient has been observed in other coral reef systems^41^ and has been attributed to the effect of territorial fishes such as farming damselfish that defend turf gardens and exclude other grazers^42^, increased rates of sedimentation in nearshore habitats that reduce quality of algal turfs^43^, and increasing productivity of algae with distance from shore^44^. In our case all three hypotheses are supported; compared to mid-lagoon reefs, fringing reefs had higher cover of *Stegastes* turf and sedimented turf as well as lower turf productivity. Therefore, in our system fringing reefs appear to be less attractive foraging habitats for grazers due to the quality and accessibility of algal turfs. As spatial scale increases, heterogeneity in abiotic and biotic factors can obscure the influence of herbivory on the benthic community^45^.

Our findings show that knowledge of spatial heterogeneity in browsing rates is useful in predicting which sites have the potential for reversal of a macroalgal state shift. Although our inferences are drawn from an experiment conducted at a small subset of sites across the gradient in browsing intensity in our system, this approach appears to be promising in identifying sites with respect to reversibility. Applying this technique more broadly in other coral reef systems will shed light on the utility of this approach in informing management. In our study, characteristics related to the macroalgae community did not appear to explain variation in browsing. Instead, browsing was most influenced by distance from deep water drop-offs. This relationship between browsing and deep water may not be universal on coral reefs, but in this case, it is likely driven by local fishing dynamics. In Moorea and generally in French Polynesia, browsers such as *Naso* (unicornfish) are highly prized for their taste and market value^39^. Although *Naso* are typically difficult to catch and demonstrate fearful behavior of humans—further indicative of the high fishing pressure they face—fishers in Moorea show high selectivity for them^36^. Free-diving spearfishers report targeting browsers on reefs near deep water as they believe it provides a spatial refuge for the fish which are much less frequently encountered in the lagoon (SI Table 1).

Caution should be exercised when linking measures of abundance to functional impact^20^. We found that estimates of herbivore biomass derived from diver surveys alone were poor predictors of herbivory rate. This decoupling between herbivore biomass and functional impact has been noted previously (e.g.,^46^), and can occur for various reasons. The grazers and browsers investigated in our study are mainly comprised of highly mobile herbivores, and therefore may not be counted in visual censuses where they feed^47^, particularly if they are harvested. Fearful behavior in response to human presence by some herbivorous fishes can result in low biomass estimates in diver surveys^48,49^ like we observed in this study for browsers (SI Table 1), which are highly prized by local spearfishers^32,38^. Another potential explanation is related to the functional dilution of herbivores as the cover of algae increases, which may result in low herbivory estimates even in areas with high herbivore biomass^16^. Therefore, we propose that monitoring both herbivore biomass *and* rates of algal removal may be the most effective method to evaluate the status of vulnerable reefs, as grazing and browsing assays are relatively rapid and cost-effective, and appear to provide reliable assessments of key trophic processes associated with coral reef resilience^50,51^. Estimating the two types of herbivory processes, in addition to standing biomass of herbivores, provides a more direct measure of the potential influence of the complementary guilds of herbivores on ecosystem functioning and reef resilience^20,32,52^.

Our results indicate grazing and browsing were spatially variable and not strongly correlated with each other across sites. Experimental work and time-series data demonstrate that reductions in grazing can lead to the proliferation of macroalgae on reefs^4,15,18^, and that browsing may be linked to the likelihood of reversing a coral-to-macroalgae state shift^13,31^. It follows that sites with low levels of grazing and browsing are likely vulnerable to shifting to and remaining trapped in a macroalgae-dominated state. By contrast, sites with high levels of both herbivory processes may be resilient due to high prevention and reversal potential. Sites characterized by high grazing and low browsing probably have moderate resilience: establishment of macroalgae is unlikely, but so is recovery of the coral state should a shift occur (i.e., a coral-macroalgae ‘phase shift’ rather than a regime shift). Interestingly, our surveys revealed that sites were not evenly distributed among these four possible herbivory regimes. There was a complete lack of low grazing-high browsing sites, which may not be surprising given the lower ratio of browsers to grazers generally in Moorea. Only 15% and 20% of sites fell into low grazing-low browsing and high grazing-high browsing regimes, respectively, whereas the majority of sites (65%) were characterized by high grazing and low browsing.

The observed pattern of spatial covariation in grazing and browsing functions could be shaped in part by patterns and preferences of fishers in the small-scale fishery of Moorea^36,38,53^ that, coupled with different life histories that make browsers more susceptible to overfishing^32^, can influence both overall biomass of herbivorous fishes and the disparity between grazers and browsers. In systems such as in Moorea where browsers are target species, the first component of resilience to be weakened through fishing is likely to be the reversibility of a coral-to-macroalgae state shift (i.e., system moves from green to yellow box in Fig. 5b). As fishing intensity continues to increase, the ability of the herbivore community to prevent a shift to macroalgae will subsequently be eroded (i.e., system moves from the yellow to the red box in Fig. 5b), and the system will remain trapped in the macroalgae state. This trajectory of reef degradation could represent the future for reefs worldwide that support small-scale fisheries in which herbivores are targeted species^13,30,34,54^. However, knowledge of spatial covariation in herbivore functions could provide a useful template for spatially-explicit management actions tailored to local conditions.

Our results indicate there likely will not be a single strategy to preserve grazing and browsing functions on reefs. Management goals will require different strategies based on key environmental factors and human pressures influencing each herbivory process. For example, managers may need to mitigate sedimentation caused by certain land-use practices to preserve grazing, while limiting fishing may be a better strategy to enhance browsing capacity. Managers may also need to apply strategies in different reef areas since grazing and browsing hot spots do not necessarily coincide in the same locations. One factor we found important in predicting the biomass of grazing and browsing herbivores alike was algal productivity, which itself can be influenced by bottom-up forcing (nutrient enrichment)^12,53^. Therefore, management could support grazers and browsers simultaneously by mitigating stressors or disturbances that alter algal productivity (e.g., nutrients, sediments) in ways that affect top-down control. Finally, management practices historically have focused on means to preserve grazing functions on coral reefs to prevent coral-to-macroalgae state shifts, which is a reasonable priority given the potential challenges in reversing such a shift. However, the frequency and intensity of disturbances to coral reefs that alone can trigger state tipping are predicted to increase. As such, greater attention should be given to enhance protection of browsers and their functional impact to reefs. To that end, our study suggests that understanding spatial covariation in grazing and browsing functions can help better target management actions to enhance resilience of coral.

## Methods

### Study site

This study was conducted in the shallow lagoons of Moorea, French Polynesia (17°30’ S, 149°50’ W) (SI Fig. 1). A barrier reef ~ 1 km offshore protects the lagoons from the open ocean except for 2 to 4 breaks produced by deep reef passes on each of the 3 sides of the island (SI Fig. 1c). Back reef habitats shoreward of the barrier reef are characterized by a short band of contiguous reef substrate that then transitions into patch reefs (bommies) surrounded by sand in the mid-lagoon. Mid-lagoon reefs are highly variable with respect to cover of coral, macroalgae, cropped turf algae, and other taxa. Directly adjacent to shore are shallow fringing reefs that can be separated from the mid-lagoon by deep channels. Water circulation within the lagoon is driven by waves forcing water over the crest of the barrier reef, through the lagoons, and out the passes^55^. Hydrodynamic circulation patterns, along with terrestrial run-off, concentrate nutrient enrichment at passes, bays, and beneath major watersheds^12^.

Along Moorea’s north shore, we selected twenty sites spread across the four lagoons that are divided by three reef passes (Fig. 1a,b and SI Fig. 1c). Ten sites were located on the fringing reef, and ten were in the mid-lagoon. At each site we quantified rates of grazing and browsing, as well as biomass and taxonomic composition of the herbivorous fish community, benthic community composition, productivity of algal turf and of macroalgae, and nutrient enrichment. To determine the distance of each site from geographic features of interest (e.g., the barrier reef crest, deep water drop-offs), we used spatial data layers produced from a previous study^53^ that mapped the inner edge of the reef crest and the coastline based on LiDAR-based digital elevation maps^56^, as well as satellite imagery provided by Google Earth Pro (Version 7.3.6.9345). For each site, we calculated the minimum distance from the reef crest using the ‘sf’ package^57^ in R (version 4.1.1). Then, we used the ruler tool in Google Earth Pro to measure the minimum distance between a site and its nearest deep-water channel, which was visually classified by having deep blue color (as opposed to visible reef substrate or sand) and being located within the lagoon.

### Quantifying spatial patterns in herbivory

We deployed assays to quantify levels of both grazing on algal turf and browsing on macroalgae at the 20 sites. For grazing, we exposed uniform pieces of turf-covered reef substrate to herbivores for 3 h between the hours of 10:00 and 16:00. Turf offerings consisted of 7 × 7 cm^2^ pieces of dead coral rubble covered by highly palatable turf (e.g., *Polysiphonia* spp.) that were collected from gardens in the lagoon that were cultivated by farming damselfish (*Stegastes* spp.). Each of these was fastened to a rack constructed of PCV-coated galvanized wire mesh that was affixed to open substrate (i.e., lacking coral or macroalgae) on the tops of patch reefs (N = 5 replicates per site). Racks at each site were placed at least 5 m apart. The percent of turf that was consumed after 3 h was estimated visually in the field by the same observer, similar to methods used in other studies^15,58^. Two trials of the grazing assay were conducted two weeks apart and all replicates at each site were pooled (N = 10 replicates per site) and then averaged to obtain the percent turf consumed, which we used as an estimate of grazing intensity. Simultaneously, we conducted browsing assays using macroalgae offerings. We attached two 15 cm-long pieces of the palatable brown macroalga, *Sargassum pacificum*, to a rack using clothes pins to hold them upright and deployed them as described above (N = 5 replicates per site). Lengths of *Sargassum* pieces were measured (cm) after 24 h (see ^58^) to determine the percent consumed of the initial 15 cm stipe; values for the two fronds deployed together were averaged for each replicate. Three trials of browsing assays were conducted two to three weeks apart and all replicates were pooled (N = 15 replicates per site) and averaged for each site to calculate the percent consumption, our estimate of browsing intensity. To explore spatial patterns of herbivory we created maps of grazing and browsing estimates from the twenty sites using the ‘sf’ package^57^ in R (version 4.1.1). Using the R statistical computing software (version 4.3.2,^59^), covariation between grazing and browsing across sites was determined using Pearson’s correlation coefficient. We used Moran’s I test to assess spatial autocorrelation in herbivory rates across the study sites.

### Quantifying herbivorous fish communities and benthic composition

The assemblage of herbivorous fishes at each site was characterized using visual surveys in which an observer on snorkel swam a 30-min timed transect, counting and visually estimating total lengths (TLs) of mobile herbivorous fishes ≥ 10 cm TL in a 5 m wide swath. The observer towed an inflatable float behind them with a Garmin GPSMap 78 handheld GPS (Olathe, Kansas, USA) to geo-reference fish counts and provide an estimate of the area covered by the survey^60^. TLs were used to estimate biomass using published species-specific relationships, and species were assigned to functional groups^61^. Based on the fish biomass estimates and the area covered by the survey (~ 500 m^2^), we calculated biomass per unit area, expressed as g/m^2^ of grazers and browsers.

Benthic composition was quantified along 50 m × 1 m long transects (N = 3 transects per site), in which the substratum was recorded at every 0.5 m (101 points per transect, 303 points per site). Substratum categories consisted of live scleractinian corals and macroalgae identified to genus, ‘other sessile invertebrates’ (mainly giant clams and sea cucumbers), rubble, sand, and three categories of turf: (1) turf inundated with sediment (hereafter ‘sedimented turf’), (2) turf growing within farmerfish (*Stegastes nigricans*) gardens (hereafter ‘*Stegastes* turf’), and (3) closely cropped turf without sediment and located outside of a *Stegastes* garden (hereafter ‘cropped turf’). Turf was considered sedimented if unconsolidated particles were visible upon inspection. The number of points for each substratum category was divided by the total 303 points per site and multiplied by 100 to estimate percent cover.

### Quantifying the nutrient environment and algal productivity

The waters surrounding Moorea are highly oligotrophic, so we used the nitrogen tissue content of a common macroalga, *Turbinaria ornata*, as a proxy for the local nutrient environment during a period of up to three months prior to *Turbinaria* collection^12,53^. *Turbinaria* responds to N pulses by storing surplus N^62^ and N tissue content can provide a time-integrated measure of N availability^63–65^. In June of 2017, a total of 10 *Turbinaria* stipes were randomly collected at each site and transported damp to the laboratory, where 10 florets were removed from each stipe 5 cm from the tip. Samples were dried in a drying oven for 4 days at 60 °C. Total N content was determined via elemental analysis using a CHN Carlo-Erba elemental analyzer (NA1500) at the University of Georgia Center for Applied Isotope Studies.

Productivity of turf in the absence of fish herbivory was measured by allowing turf to colonize and grow for 21 days on 2.5 cm^2^ unglazed terra cotta tiles in herbivore-exclusion cages (N = 8 caged tiles per site). Herbivore exclusion cages were 10 × 10 × 10 cm galvanized mesh, with mesh size of 2.5 cm. After the deployment period, tiles were brought to the lab, and turf was removed and processed to obtain ash-free dry weight (AFDW). The mean value of AFDW for each site provided an estimate of turf productivity (g accumulated 3 wk^−1^). Macroalgal productivity was quantified by allowing juvenile *Turbinaria*, and other colonizing macroalgae, to grow protected from herbivores for 8 weeks. We collected reef substratum with attached juvenile *Turbinaria* from the mid-lagoon and removed all other algae so only juvenile *Turbinaria* (≤ 3 cm length) of uniform size and density remained. The substrates containing *Turbinaria* were attached to the bottom of herbivore-exclusion cages at each site (N = 8 per caged replicates per site). After two months, *Turbinaria* and any other colonizing macroalgae were removed and damp weighed. Weights of replicates were pooled at a given site to provide an estimate of macroalgal productivity (g accumulated 8 wk^−1^).

### Relationships between spatial variation in herbivory, herbivore biomass, and environmental attributes

Covariation between grazing rate and the biomass of grazing fishes from our fish counts across the twenty sites was determined using Pearson’s correlation coefficient (version 4.3.2;^59^). The same analysis was conducted to assess the relationship between browsing rate and the biomass of browsing fishes across sites. We then utilized principal component analysis (PCA) in R (version 4.3.2;^59^) to explore the degree to which environmental factors could explain observed spatial patterns in browsing and grazing, specifically, the ‘hot spots’ (high grazing or browsing activity) and ‘cold spots’ (low activity) in the lagoon. A subset of sites that reflected the highest (hot spots) and lowest (cold spots) levels in one of the herbivory processes was selected for this analysis, utilizing an additional criterion that the level of the second herbivory process was similar among the group. To explore variation in grazing we chose sites that exhibited the highest or lowest grazing, but that had similar (low) levels of browsing. This selection process resulted in five high grazing sites (i.e., grazing hot spots) and three low grazing sites (i.e., grazing cold spots) (Fig. 3 bottom right and left corners). The full range of browsing only occurred among sites with moderate to high levels of grazing. Four high browsing sites (i.e., browsing hot spots) and five low browsing sites (i.e., browsing cold spots), all of which had high levels of grazing were chosen for analysis (Fig. 3 right top and bottom corners).

Explanatory variables for the PCA analyses included both spatial factors as well as variables that reflected the amount and productivity of the food resources of the fishes and therefore could reflect the intensity of browsing or grazing. Location in the lagoon can be important predictors of fish spatial patterns of abundance and activity, and each site’s distance from the reef crest and from deep water drop-offs were included as predictor variables. The amounts of algal cover and productivity impact the distribution and feeding behavior of herbivorous fishes^66^. For example, turf can vary in palatability or accessibility to grazing herbivores due to sediment load^43,67^ or whether it is guarded by territorial farming damselfish (*Stegastes* spp.), with sedimented turf and turf within farmerfish gardens comprising less preferred feeding substrates than unsedimented and undefended turf. Therefore, turf productivity and cover of different types of turf (turf, sedimented turf, turf inside *Stegastes* gardens) were included in the PCA for grazing, and cover and productivity of macroalgae for browsing. The analysis was based on a correlation matrix of these data.

### Predicting variation in herbivore biomass using environmental attributes

To explore whether environmental attributes associated with hot spots and cold spots in herbivory also predict variation in the biomass of herbivores, we utilized the best subset selection approach, which is an exploratory model building regression analysis. Using predictor variables from the grazing PCA analysis, we utilized the R package “leaps” (Version 3.1) to test all possible combinations of predictors in a multiple linear regression to explain variation in grazer biomass, and then selected the best model according to lowest AIC score with the R package “AICcmodavg” (Version 2.3). The same procedure was done for browsers using predictors from the browsing PCA.

### Linking spatial variation in herbivory to the resilience of coral

Because of a general lack of data that link browsing intensity and reversibility of state shifts to macroalgae, we explored this relationship using a field experiment. Macroalgae-dominated communities were exposed to herbivores along a natural gradient in browsing intensity to evaluate whether reversal potential was related to variation in browsing. We selected five sites that represented the gradient in browsing intensity we observed in our short-term herbivory assays described above. An advantage of a gradient experiment is that it can better identify non-linear relationships compared to a replicated design. At each site, we deployed macroalgae communities that mimicked patch reefs that had undergone a shift to macroalgal dominance. Each community was constructed by chiseling off reef substrate with attached *Turbinaria*, which was then assembled into a community that reflected the size and density of macroalgae-dominated patch reefs within the lagoon. This assemblage was then epoxied to a cinderblock and the density of adult *Turbinaria* stipes recorded. The cinderblock macroalgal community (N = 6 replicates per site) was then deployed at a depth within 2–5 m and left exposed to herbivores for 10 days. At the end of the experiment, final adult *Turbinaria* density was recorded. The percent change in density of adult *Turbinaria* was calculated for each replicate using the equation (*Final* – *Initial*) / *Initial* × 100. Replicate values were then averaged for each site and used as a proxy for reversibility of a coral-to-macroalgae state shift.

Lastly, we were interested in whether environmental features associated with variation in browsing were also good predictors of reversibility. Covariation between reversibility and distance from deep water (log-transformed) for the five sites was determined using Pearson’s correlation coefficient (version 4.3.2;^59^).

## Supplementary Information

Supplementary Information.

## Data Availability

All data sets and code utilized for this research are publicly available from the Environmental Data Initiative (EDI) Data Portal: https://doi.org/10.6073/pasta/0a0ccafc1337307182e4ce0c348a0d73.
