# Supplementary material for: Patterns and implications of spatial covariation in herbivore functions on resilience of coral reefs
Source: Sci Rep. 2025 Jan 7;15:1176. doi: 10.1038/s41598-024-83672-1 (PMC11707016; doi:10.1038/s41598-024-83672-1)
Supplement: Supplementary file 1 — Supplementary Information. [file 41598_2024_83672_MOESM1_ESM.docx]

**Supplementary Information**

**Journal name:** Scientific Reports

**Manuscript title:** Patterns and Implications of Spatial Covariation in Herbivore Functions on Resilience of Coral Reefs

**Authors:** Dana T. Cook, Sally J. Holbrook and Russell J. Schmitt

**SI Table 1.** Total and proportional biomass and abundance of herbivorous fish taxa from visual surveys conducted by divers at the 20 study sites in 2017. Biomass is measured in units of g/m^2^ and abundance is number of individuals.

| Species | Herbivore Group | Total Biomass | Total Abundance | Proportional Biomass | Proportional Abundance |
| --- | --- | --- | --- | --- | --- |
| Acanthurus guttatus | Grazer | 0.347 | 3 | 0.0005 | 0.0005 |
| Acanthurus lineatus | Grazer | 0.976 | 5 | 0.0014 | 0.0009 |
| Acanthurus nigricans | Grazer | 0.098 | 1 | 0.0001 | 0.0002 |
| Acanthurus nigricauda | Grazer | 8.651 | 35 | 0.0123 | 0.0063 |
| Acanthurus nigrofuscus | Grazer | 104.841 | 1431 | 0.1496 | 0.2576 |
| Acanthurus nigroris | Grazer | 2.380 | 18 | 0.0034 | 0.0032 |
| Acanthurus pyroferus | Grazer | 0.553 | 7 | 0.0008 | 0.0013 |
| Arothron meleagris | Grazer | 1.014 | 2 | 0.0014 | 0.0004 |
| Chlorurus sordidus | Excavator | 177.459 | 1167 | 0.2531 | 0.2101 |
| Leptoscarus vaigiensis | Browser | 3.178 | 9 | 0.0045 | 0.0016 |
| Naso lituratus | Browser | 3.046 | 24 | 0.0043 | 0.0043 |
| Naso unicornis | Browser | 0.918 | 1 | 0.0013 | 0.0002 |
| Scarus altipinnis | Scraper | 6.431 | 15 | 0.0092 | 0.0027 |
| Scarus globiceps | Scraper | 3.004 | 5 | 0.0043 | 0.0009 |
| Scarus oviceps | Scraper | 22.676 | 146 | 0.0323 | 0.0263 |
| Scarus psittacus | Scraper | 274.423 | 1701 | 0.3915 | 0.3062 |
| Siganus spinus | Browser | 12.592 | 126 | 0.0180 | 0.0227 |
| Zebrasoma scopas | Grazer | 78.365 | 858 | 0.1118 | 0.1545 |
| Zebrasoma veliferum | Grazer | 0.082 | 1 | 0.0001 | 0.0002 |

**SI Table 2.** Summary statistics for multiple linear regressions predicting variation in the biomass of grazers (left) and browsers (right).

|  | $\frac{Dependent variable:}{Grazer biomass}$ |  |  | $\frac{Dependent variable:}{Biomass biomass}$ |
| --- | --- | --- | --- | --- |
| Sedimented turf cover | 0.262 |  | Macroalgae productivity | 0.009** |
|  | (0.157) |  |  | (0.003) |
| Turf productivity | 629.629* |  | Distance from reef crest | -0.001 |
|  | (312.928) |  |  | (0.0004) |
| Constant | 11.154 |  | Constant | 0.660 |
|  | (9.542) |  |  | (0.403) |
| Observations | 20 |  | Observations | 20 |
| R^2^ | 0.318 |  | R^2^ | 0.425 |
| Adjusted R^2^ | 0.237 |  | Adjusted R^2^ | 0.357 |
| Residual Std. Error | 12.562 (df = 17) |  | Residual Std. Error | 0.384 (df = 17) |
| F Statistic | 3.956** (df = 2; 17) |  | F Statistic | 6.280*** (df = 2; 17) |
| *Note:* | *p<0.1; **p<0.05; ***p<0.01 |  | *Note:* | *p<0.1; **p<0.05; ***p<0.01 |


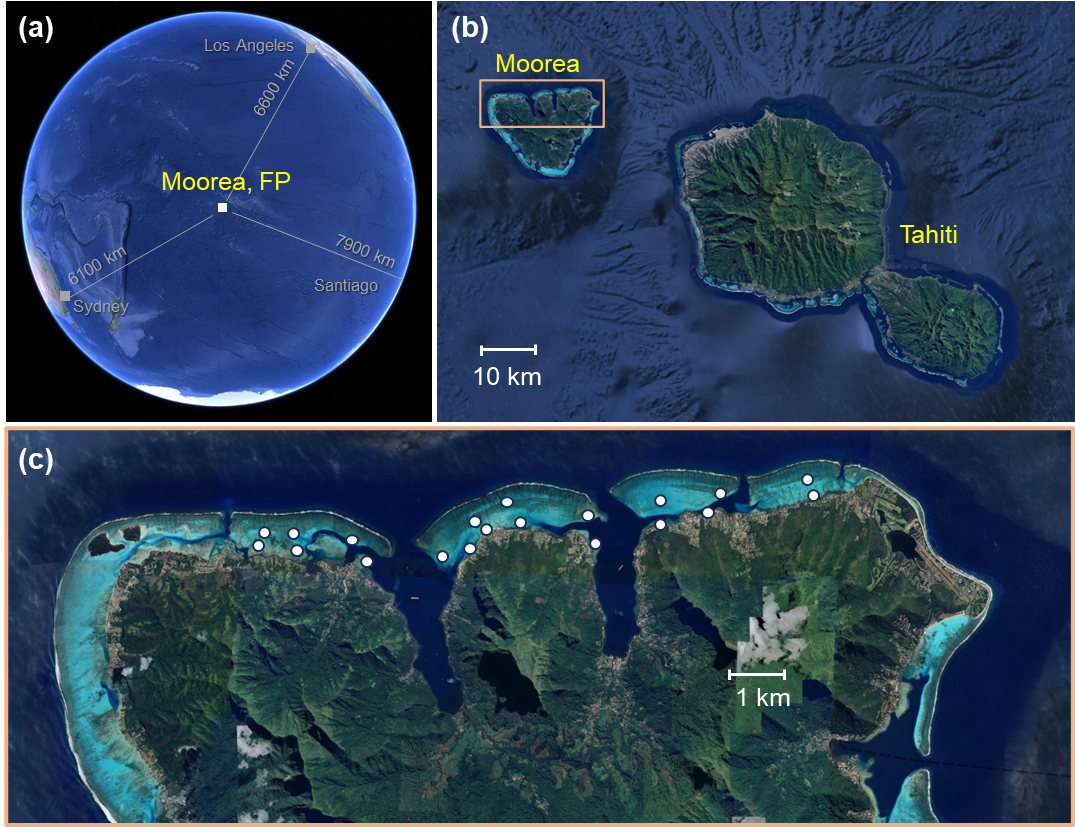


**Supplemental Figure 1.** **(a)** Location of Moorea, French Polynesia in the central South Pacific Ocean, **(b)** situated in the leeward Society Islands ~ 15 km west of Tahiti. **(c)** The northern portion of Moorea (orange rectangle in (b)) showing the 20 sampling stations distributed in mid-lagoon and fringing reef habitats over ~ 10.5 km stretch of the north shore. All 3 images courtesy of Google Earth Pro (v. 7.3.6) with imagery dates of 12/13/2015.

**
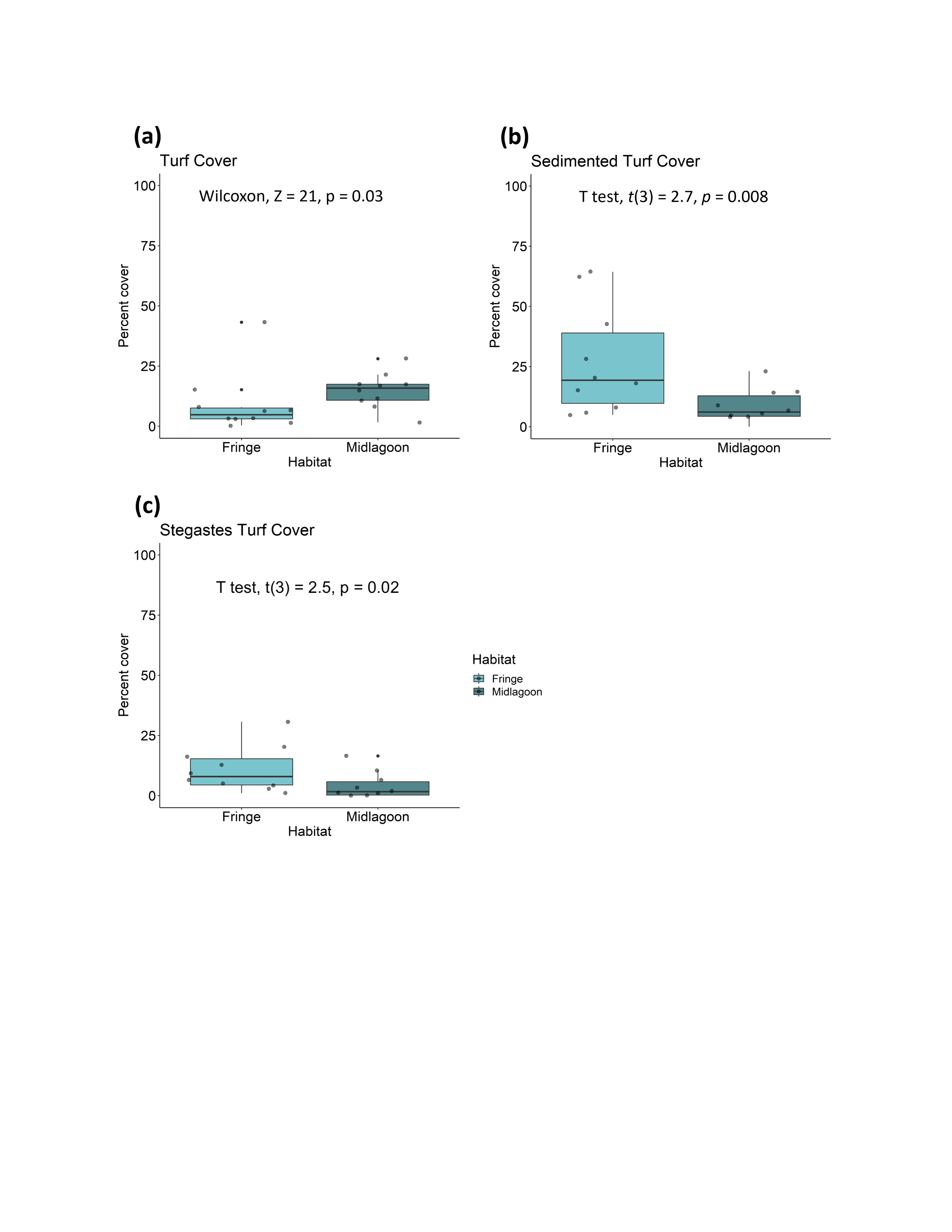
**

**Supplemental Figure 2.** Boxplots showing differences in turf community composition between mid-lagoon and fringing reef habitats (N = 10 sites per habitat). Cover of three types of turf are shown: **(a)** turf, **(b)** sedimented turf, and **(c)** *Stegastes* turf. Grey circles indicate the average percent cover of turf at a site.

**
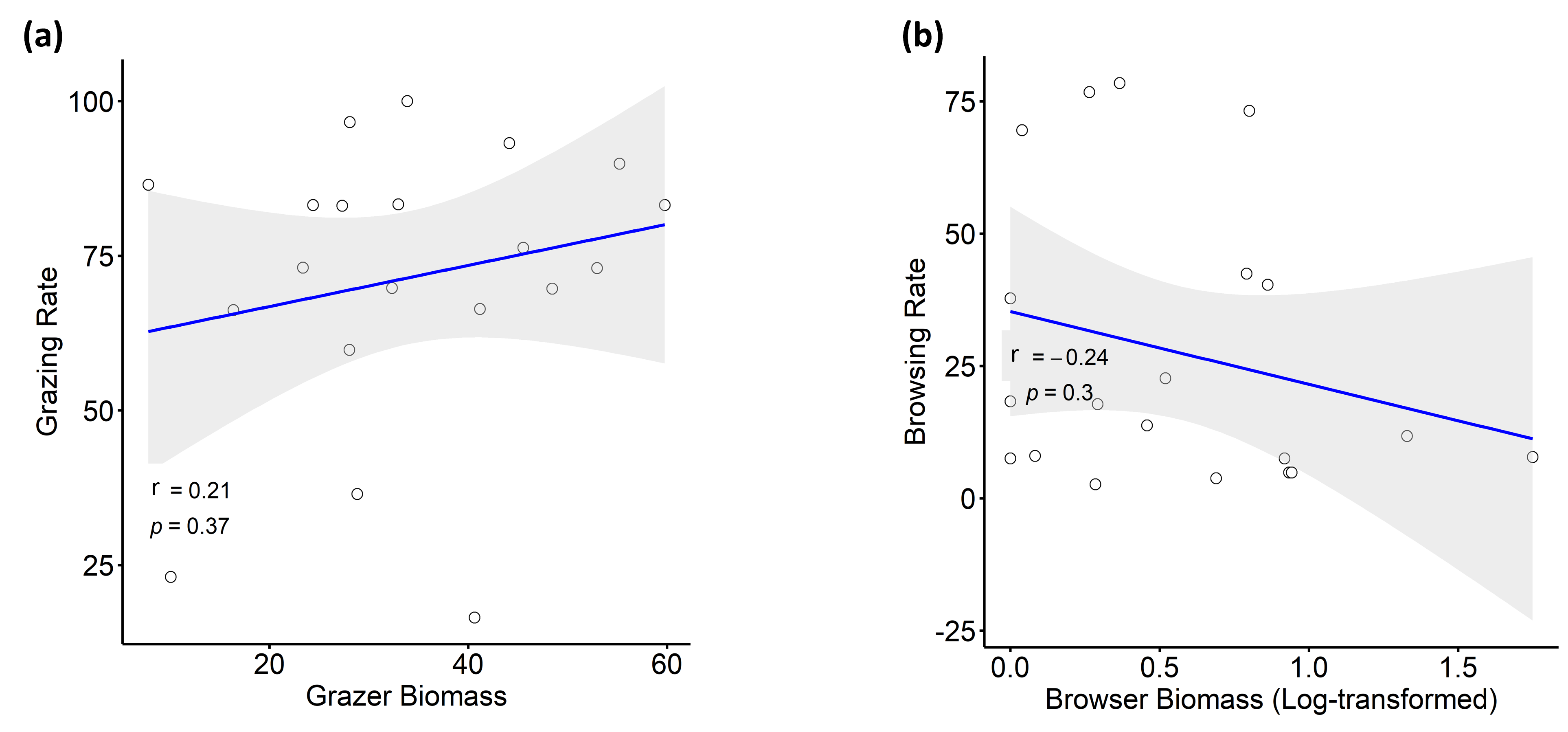
**

**Supplemental Figure 3.** Relationships between **(a)** grazing rate and grazer biomass and **(b)** browsing rate and browser biomass at the twenty sites. Circles are site averages of herbivore biomass and grazing or browsing rates.


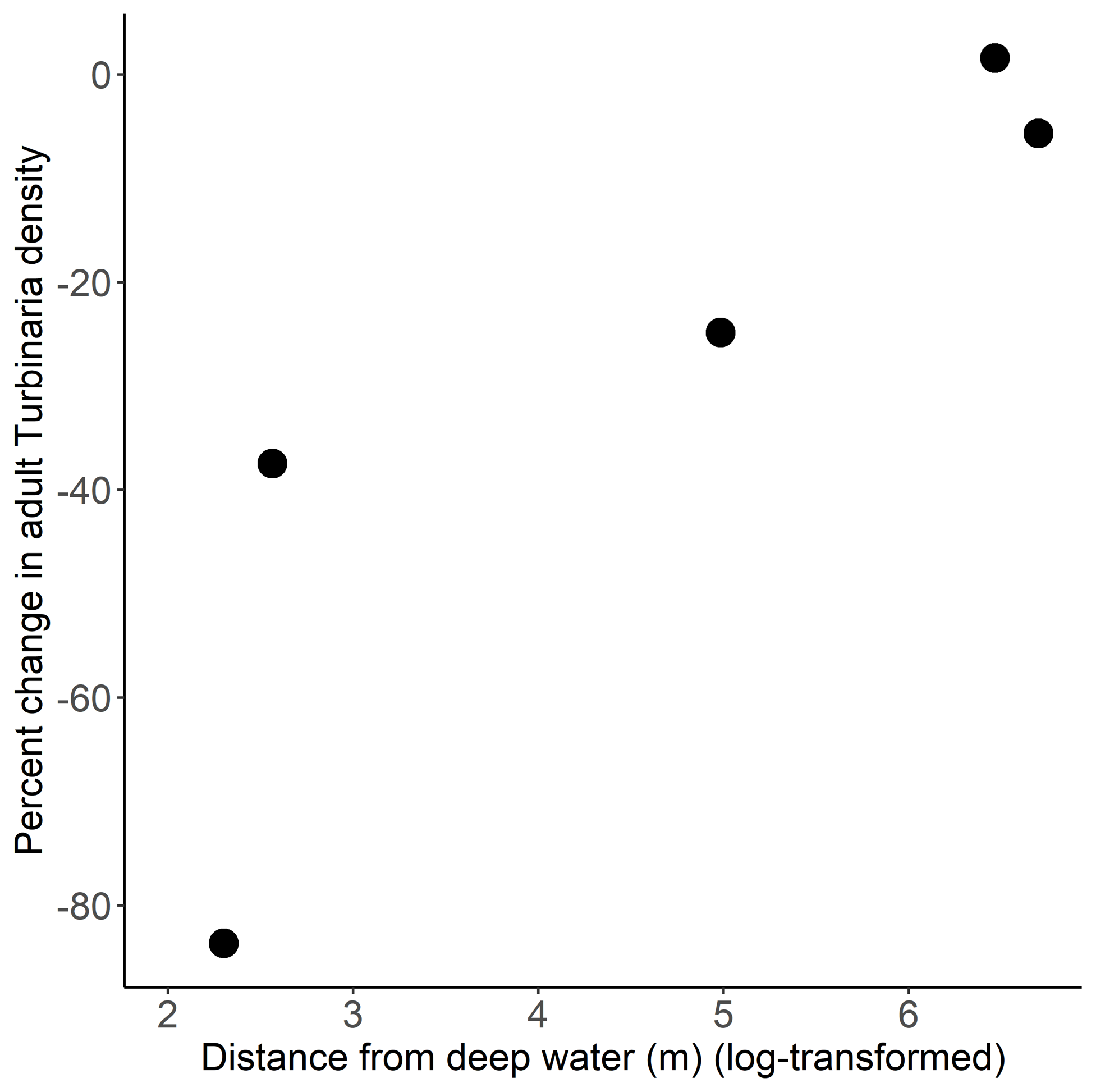


**Supplemental Figure 4.** Relationship between removal of macroalgae communities (i.e., reversibility of coral-to-macroalgae state shifts) and distance from deep water (log-transformed).
